# The Alleviating Effect of Arginine on Ethanol Stress in *Wickerhamomyces anomalus*

**DOI:** 10.3390/jof11070499

**Published:** 2025-07-02

**Authors:** Yinfeng Li, Yujie Wang, Shuangyan Liu, Guilan Jiang, Mingzheng Huang, Xiaozhu Liu

**Affiliations:** 1 College of Food and Pharmaceutical Engineering, Guizhou Institute of Technology, Guiyang 550000, China; liyinfeng614@126.com (Y.L.);; 2 College of Bioscience and Biotechnology, Hunan Agricultural University, Changsha 410128, China

**Keywords:** *Wickerhamomyces anomalus*, ethanol stress, arginine, transcriptomics, metabolomics, nitric oxide

## Abstract

Yeast cells are passively exposed to ethanol stress during alcoholic fermentation, ultimately impairing cell viability and reducing fermentation efficiency. Arginine, a versatile amino acid, plays a crucial role in regulating cellular responses to various stresses. This study aimed to explore the underlying mechanism by which arginine protects *Wickerhamomyces anomalus* against ethanol stress. The effects of arginine supplementation (5 mM) under ethanol stress (9% *v*/*v*) on cell survival, reactive oxygen species (ROS) production, cellular and mitochondrial membrane integrity, and nitric oxide synthesis were investigated using fluorescent staining methods. Furthermore, differentially expressed genes (DEGs) and metabolites (DEMs) were identified through transcriptomics and metabolomics analyses. The results demonstrated that exogenous arginine enhanced cell survival, reduced ROS levels, maintained cellular and mitochondrial membrane integrity, stimulated nitric oxide production, and modulated gene expression and metabolic pathways involved in pyruvate metabolism, yeast meiosis, fatty acid degradation, glycerophospholipid metabolism, and the biosynthesis of various secondary metabolites. These findings provide intriguing insights into the mechanistic role of arginine in enhancing the tolerance of *W. anomalus* to ethanol stress, and broaden its application in the fermentation industry for alcoholic beverages.

## 1. Introduction

Yeasts have been extensively utilized by humans for millennia as effective microbial cell factories for the production of alcoholic beverages [1]. However, during the process of ethanol fermentation, yeast cells are inevitably exposed to multiple adverse environmental stresses, including temperature extremes (both high and low), acidity, osmotic pressure, ethanol, and oxidative stress [2]. Among these stressors, ethanol is considered the primary stressor due to its constant accumulation during the fermentation process. Evidence indicates that ethanol stress can elicit bursts of reactive oxygen species (ROS), disrupting the intracellular oxidation–reduction balance and causing denaturation of cellular macromolecules such as proteins, DNA, and lipids [3]. Furthermore, ethanol molecules can compromise the integrity of cellular structures, including the cell wall, cell membrane, and organelles [4]. Consequently, gene expression and metabolic processes are perturbed, leading to impaired cell growth and viability, and ultimately, reduced fermentation efficiency under ethanol stress conditions.

In response to ethanol stress, yeast cells exhibit complex adaptive mechanisms that involve alterations in their amino acid metabolism [5]. Various amino acids, notably isoleucine, methionine, phenylalanine, tryptophan, proline, and arginine, have been implicated in the ethanol stress response of *Saccharomyces cerevisiae* and *Kluyveromyces marxianus* [5,6,7]. Our recent study, employing advanced transcriptomics and metabolomics approaches, has provided insights into the metabolic adaptations of *Wickerhamomyces anomalus* under ethanol stress conditions [8]. Specifically, we found that ethanol stress modulates the metabolic expression of arginine, aspartic acid, and glutamate in *W. anomalus*. These findings suggest that these amino acids and their associated metabolic pathways may play pivotal regulatory roles in the yeast’s response to ethanol stress. By understanding how these amino acids are metabolized and regulated under stress conditions, we can gain deeper insights into the adaptive mechanisms of *W. anomalus* and potentially identify strategies to enhance its tolerance to ethanol stress, thereby improving the efficiency and productivity of fermentation processes.

Arginine, a versatile basic amino acid, plays a multitude of roles within living cells. Besides being a fundamental component of proteins and serving as a precursor for the biosynthesis of proline, polyamines, and nitric oxide, arginine also plays a crucial part in regulating various stress responses [9]. Exogenous arginine application has proven effective for alleviating diverse plant stresses. For instance, it mitigates oxidative damage in water-stressed tomatoes [10], enhances physiological and metabolic functions in drought-stressed wheat [11], and improves salt stress resilience in sunflowers [12].

In yeast species, the induction of arginine biosynthetic genes in response to ROS generated by host stress has been observed in *Candida albicans* [13]. Further research has revealed that arginine functions as a novel compatible solute in *Candida glabrata*, enhancing its resistance to hyperosmotic conditions by decreasing arginine degradation and increasing its biosynthesis [14]. Similarly, in *S. cerevisiae*, arginine offers protection against ethanol stress by maintaining cellular structural integrity and reducing ROS levels [7]. Despite these advancements, the specific role of arginine in the ethanol stress response of *W. anomalus* remains incompletely understood.

In this study, the impact of arginine on the ethanol stress response in *W. anomalus* was investigated by examining cell survival and the production of ROS and nitric oxide, and assessing the integrity of both cellular and mitochondrial membranes. Furthermore, advanced transcriptomics and metabolomics approaches were employed to gain a comprehensive understanding of how exogenous arginine supplementation affects the yeast’s metabolic and genetic landscape under ethanol stress. The present study offers intriguing insights into the mechanistic role of arginine in enhancing the tolerance of *W. anomalus* to ethanol stress, which may be beneficial for the application of *W. anomalus* in the production of *Baijiu* and fruit wines.

## 2. Materials and Methods

### 2.1. Yeast Strains and Culture Conditions

The yeast strain *W. anomalus* C11, previously isolated from the fruit of *Rosa roxburghii* Tratt (a perennial shrub belonging to the Rosaceae family), was utilized in this study [15]. To revive the cryopreserved *W. anomalus* strain, it was streaked onto yeast extract peptone dextrose (YEPD) agar medium, composed of 1% yeast extract, 2% peptone, 2% glucose, and 2% agar. The plates were then incubated statically at 28 °C for 72 h. Subsequently, the activated cells were stored at 4 °C for future use.

### 2.2. Instruments and Equipment

The laminar airflow workstation (VD850) was produced by Jinan Senya Experimental Instruments Co., Ltd. (Jinan, China). The fluorescence microscope (BX51) was produced by Olympus Corporation (Tokyo, Japan). A centrifugal machine was produced by Hunan Kaicheng Instrument and Equipment Co., Ltd. (Changsha, China). The fluorescence spectrophotometer (F-4700) was produced by Hitachi Limited (Tokyo, Japan). The biochemical incubator (DPH-420) was produced by Tianjin Tiantai Instrument Co., Ltd. (Tianjin, China).

### 2.3. Ethanol Stress and Exogenous Arginine Supplementation

Log-phase *W. anomalus* cells were prepared by culturing them in YEPD broth at 28 °C with shaking at 160 rpm for 8 h. These cells were then divided into three groups: ethanol, ethanol + arginine, and control. Cells in the ethanol group were directly exposed to 9% (*v*/*v*) ethanol, as this concentration represents the maximum tolerance threshold of the strain [7]. At 12% ethanol concentration, most cells died. Cells in the ethanol + arginine group were simultaneously exposed to 9% (*v*/*v*) ethanol and supplied with 5 mM of arginine. Cells in the control group were cultivated in YEPD broth without any additional treatment. The start of the treatment was designated as 0 h, and all groups were incubated for an additional 6 h. Post-treatment, cells were harvested for the analysis of survival, ROS levels, membrane integrity (both cellular and mitochondrial), nitric oxide content, and transcriptomic and metabolomic profiling.

### 2.4. Survival Analysis

Survival of each group was evaluated using a spot analysis method. Briefly, cells from each group were harvested by centrifugation at 4000× *g* for 10 min, washed, and resuspended in sterile distilled water. Subsequently, the cell suspensions were serially diluted to concentrations of 10^0^, 10^−2^, and 10^−4^. Aliquots of each dilution were spread onto YEPD agar plates and incubated at 28 °C for 36 h. Colony formation was then observed and photographed using a digital camera to assess the survival of the cells in each group.

### 2.5. ROS Production Detection

Intracellular ROS levels, specifically hydrogen peroxide (H_2_O_2_) and superoxide anion (O_2_^·-^), were quantified using fluorescent probes: 2′,7′-dichlorodihydrofluorescein diacetate (DCFH-DA, Ex/Em = 488/525 nm) and dihydroethidium (DHE, Ex/Em = 300/610 nm), respectively [16]. Following treatment, cells from each group were collected by centrifugation at 4000× *g* for 10 min, rinsed with phosphate-buffered saline (PBS, pH 7.4), and incubated with DCFH-DA (10 μM) or DHE (4 μM) for 30 min. The cells were then washed three times with PBS (pH 7.4). Fluorescence signals were qualitatively analyzed using a fluorescence microscope (Olympus BX51, Olympus, Tokyo, Japan) and quantitatively assessed using a fluorescence spectrophotometer (Hitachi F-4700, HITACHI, Tokyo, Japan).

Additionally, the production of O_2_^·−^ was monitored using p-nitro-blue tetrazolium chloride (NBT, 50 μM) for 15 min. The stained cells were centrifuged at 4000× *g* for 10 min, decolorized with anhydrous ethanol for 15 min, and then dissolved in a solution containing potassium hydroxide (2 M) and dimethyl sulfoxide (DMSO). The resulting color was observed under a microscope, and the optical density was measured at 630 nm using a microplate reader.

### 2.6. Cellular and Mitochondrial Membrane Integrity Assessment

The integrity of the cellular membrane was evaluated using propidium iodide (PI), a fluorescent dye that is impermeable to intact cell membranes but readily enters cells with compromised membranes [17]. Once inside, PI binds to DNA and emits fluorescence. Similarly, the integrity of the mitochondrial membrane was assessed by measuring mitochondrial membrane potential using rhodamine 123. This dye selectively accumulates in the mitochondrial matrix and fluoresces in response to the transmembrane potential. However, disruption of the mitochondrial membrane and loss of potential result in the release of rhodamine 123, leading to a decrease in cellular fluorescence.

Cells from each group were collected by centrifugation, washed with PBS (pH 7.4), and stained with either 20 μM PI (Ex/Em = 536/635 nm) or 2 μM rhodamine 123 (Ex/Em = 510/530 nm) in the dark at room temperature for 10 min. Fluorescent images were captured using a fluorescence microscope, and the fluorescence intensities were quantitatively analyzed using a fluorescence spectrophotometer.

### 2.7. Nitric Oxide Synthesis Detection

Intracellular nitric oxide levels were assayed using the fluorescent probe 3-amino,4-aminomethyl-2′,7′-difluorescein diacetate (DAF-FM DA) [18]. This probe readily diffuses into cells, where it undergoes hydrolysis by intracellular esterases to form DAF-FM. Subsequently, DAF-FM reacts specifically with nitric oxide to produce a fluorescent signal. After harvesting, the cells were washed once with PBS (pH 7.4) to remove any extracellular contaminants. They were then incubated with 2 μM DAF-FM DA (Ex/Em = 495/515 nm) at 37 °C for 20 min to allow for sufficient intracellular conversion and reaction with nitric oxide. Following incubation, the cells were rinsed three times with PBS (pH 7.4) to remove any unbound probe. Fluorescent images were captured using a fluorescence microscope, and the fluorescence intensities were quantitatively measured using a fluorescence spectrophotometer, both according to the manufacturer’s protocols.

### 2.8. RNA Extraction, Transcriptome Sequencing, and Bioinformatics Analysis

Total RNA was extracted from cells of the control, ethanol, and ethanol + arginine groups using TRIzol reagent (Invitrogen, Waltham, MA, USA) following the manufacturer’s protocol. To eliminate genomic DNA contamination, DNase I (Takara, Osaka, Japan) was employed. Subsequently, a transcriptome library was prepared using 1 μg of total RNA with the TruSeq^TM^ RNA Sample Preparation Kit (Illumina, San Diego, CA, USA). During library preparation, cDNA fragments of approximately 300 bp were size-selected on a 2% Low Range Ultra Agarose gel and amplified by PCR (15 cycles) using Phusion DNA polymerase (NEB, Ipswich, MA, USA). The resultant paired-end RNA-seq library was sequenced on the high-throughput Illumina HiSeq X Ten/NovaSeq 6000 platform (Illumina, San Diego, CA, USA).

To identify differentially expressed genes (DEGs), a fold change threshold of >2 and a statistical significance threshold of *p* value < 0.05 were applied. These DEGs were then classified and subjected to Kyoto Encyclopedia of Genes and Genomes (KEGG) enrichment analysis using the Majorbio Cloud Platform (https://cloud.majorbio.com). This analysis was conducted according to the manufacturer’s protocol and the methodology described by Li et al [8].

### 2.9. Non-Targeted Metabolomics Analysis

Non-targeted metabolomics analysis was conducted by Majorbio Biotech (Shanghai, China) utilizing liquid chromatography–mass spectrometry (Vanquish Horizon system, Thermo Scientific, Waltham, MA, USA). For this analysis, 50 mg of cells from the control, ethanol, and ethanol + arginine groups were precisely weighed. Metabolites were extracted using 400 µL of a methanol:water solution (4:1, *v*/*v*), which contained 0.02 mg/mL of L-2-chlorophenylalanine as an internal standard to ensure the accuracy and reproducibility of the analysis. The extraction mixture was chilled at −20 °C, vortexed vigorously for 30 s, and then sonicated at a frequency of 40 kHz for 30 min at 5 °C to ensure complete extraction. Following centrifugation at 13,000× *g* for 15 min, the supernatant containing the extracted metabolites was carefully collected for LC-MS analysis. Chromatographic separation and mass spectrometric conditions were set according to the methodology described by Li et al. [8].

Raw data were processed using Progenesis QI 2.3 software (Waters, Milford, MA, USA) for peak detection, alignment, and generation of a comprehensive data matrix, which included retention time, mass-to-charge ratio (*m*/*z*), and peak intensity. To identify differentially expressed metabolites (DEMs), a threshold of variable importance in projection (VIP) > 1 and a statistical significance threshold of *p* < 0.05 were employed. These DEMs were annotated and subjected to Kyoto Encyclopedia of Genes and Genomes (KEGG) enrichment analysis on the Majorbio Cloud Platform, adhering strictly to the manufacturer’s protocol.

### 2.10. Statistical Analysis

The results were expressed as the mean ± standard deviation. Univariate analysis of variance (ANOVA) of the data and the significance of the difference test was performed using SPSS 21.0 software. *p* < 0.05 was considered statistically significant.

## 3. Results

### 3.1. Arginine Enhanced the Survival of Ethanol-Stressed W. anomalus

**Figure 1:**
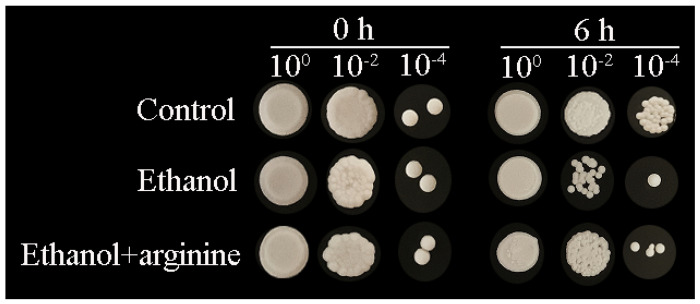
Arginine enhanced the survival of ethanol-stressed *W. anomalus*.

Exogenous arginine was supplemented to investigate its impact on the survival of *W. anomalus* subjected to ethanol stress during the logarithmic growth phase. Initially, as illustrated in Figure 1, the survival rates among all groups were comparable at the onset of ethanol treatment (0 h). However, a significant decline in survival was observed after 6 h of exposure to ethanol. Notably, the exogenous supplementation of arginine partially mitigated this decrease in cell viability, thereby enhancing the survival of *W. anomalus* under ethanol stress. These findings suggest that arginine may confer protection to *W. anomalus* against the adverse effects of ethanol stress.

### 3.2. Arginine Reduced ROS Levels in Ethanol-Stressed W. anomalus

**Figure 2:**
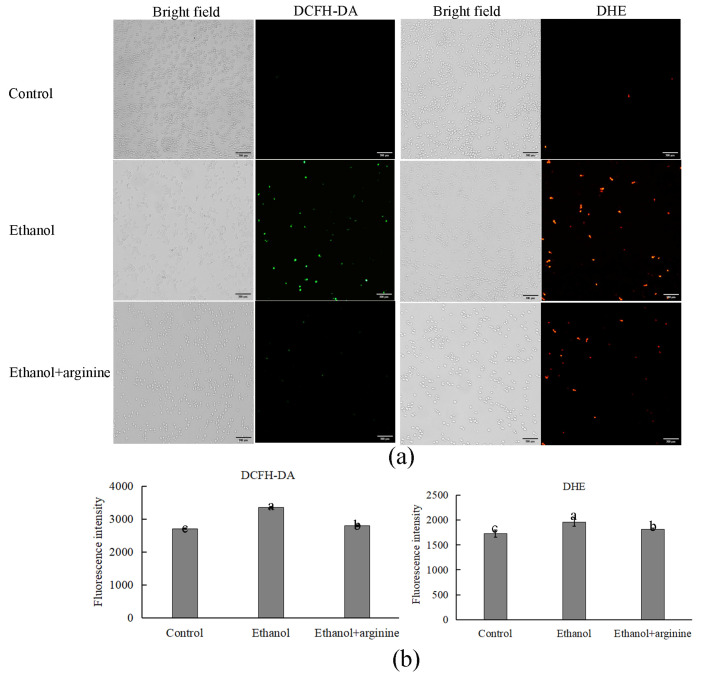
Arginine suppressed ROS production in ethanol-stressed *W. anomalus*. (**a**) Fluorescence images of each group; (**b**) fluorescence intensity of each group. Different lowercase letters indicate significant differences (*p* < 0.05). Scale bar = 300 μm.

To explore the protective mechanism of arginine in *W. anomalus* under ethanol stress, the levels of ROS with or without arginine supplementation were investigated. The fluorescent images are shown in Figure 2a. Ethanol treatment led to a marked increase in ROS levels, specifically in the forms of H_2_O_2_ (detected by DCFH-DA) and O_2_^.−^ (detected by DHE), indicating that ethanol stress induced ROS accumulation in *W. anomalus*. In contrast, the production of both H_2_O_2_ and O_2_^.−^ was significantly diminished in the group that received ethanol along with exogenous arginine supplementation. This suggests that arginine alleviated ROS production in *W. anomalus* subjected to ethanol stress.

The fluorescence intensity data for each group, as depicted in Figure 2b, aligns with the observations from the fluorescent images. Furthermore, NBT staining for O^2.−^ provided additional confirmation that arginine partially suppressed the ROS burst in *W. anomalus* under ethanol stress (Figure S1).

### 3.3. Arginine Preserved the Integrity of Cellular and Mitochondrial Membranes in Ethanol-Stressed W. anomalus

**Figure 3:**
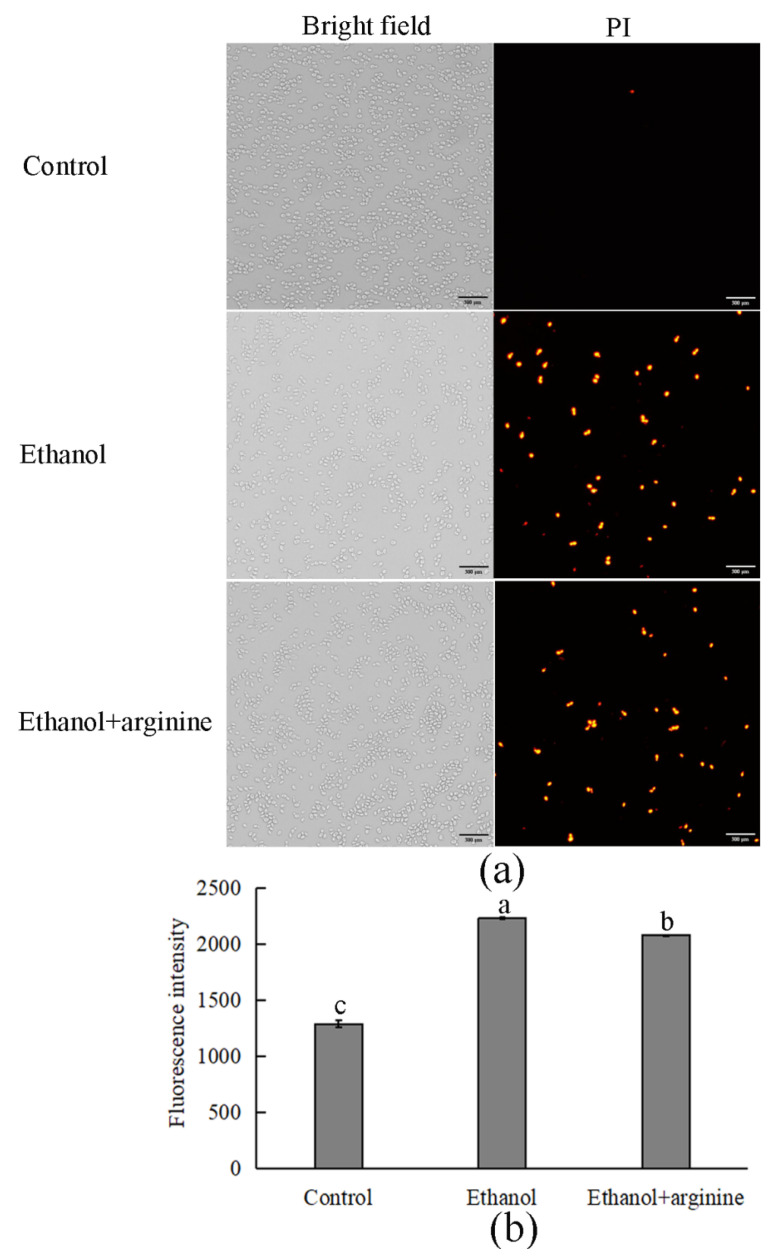
Arginine preserved cell membrane integrity in ethanol-stressed *W. anomalus*. (**a**) Fluorescence images of each group; (**b**) fluorescence intensity of each group. Values of the distinct lowercase letters indicate significant differences (*p* < 0.05). Bar = 300 μm.

Evidence has demonstrated that the cell membrane is a primary target of damage induced by ethanol [6,19]. Consequently, the fluorescent probe PI was used to evaluate the integrity of the cell membrane across different groups. In the control group, minimal fluorescence was observed, indicating intact membranes. Conversely, the ethanol group exhibited significantly heightened fluorescence (Figure 3a), suggesting membrane damage. Notably, upon the introduction of arginine, the fluorescence intensity diminished, indicative of enhanced membrane integrity (as shown in Figure 3a and quantified in Figure 3b).

**Figure 4:**
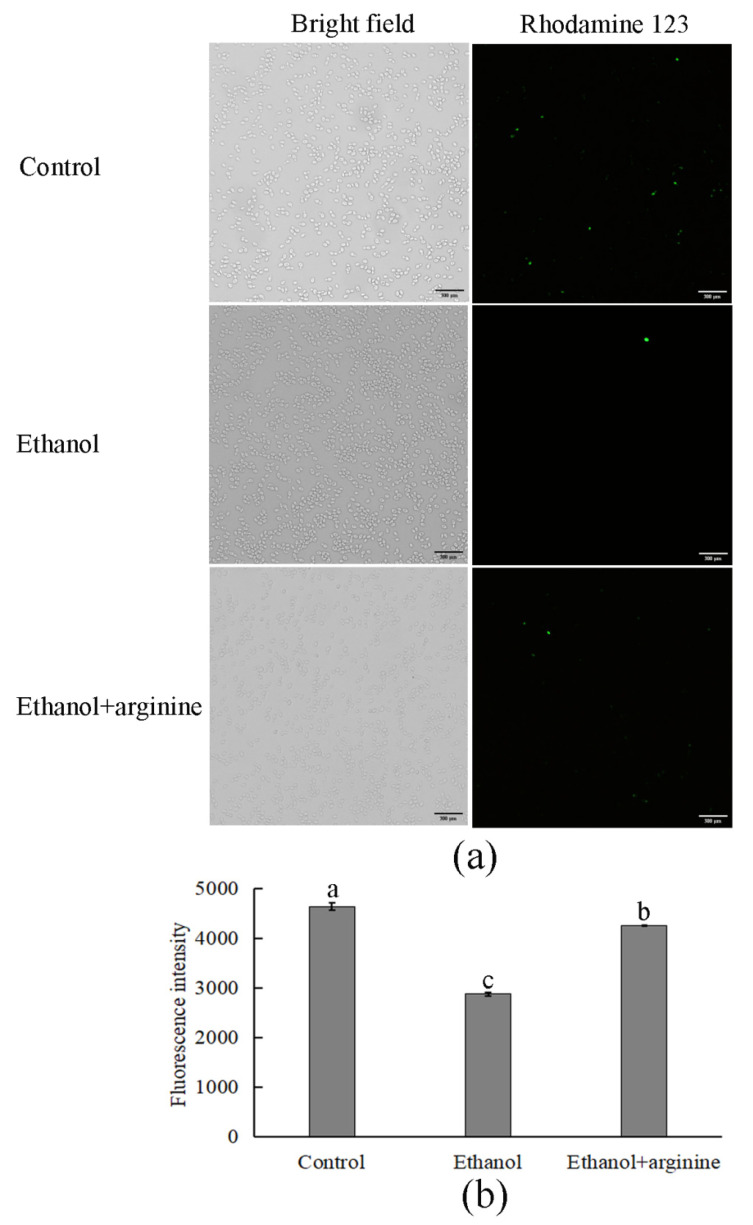
Arginine preserved mitochondrial membrane integrity in ethanol-stressed *W. anomalus*. (**a**) Fluorescence images of each group; (**b**) fluorescence intensity of each group. Different lowercase letters indicate significant differences (*p* < 0.05). Scale bar = 300 μm.

The integrity of the mitochondrial membrane was further assessed using the fluorescent dye rhodamine 123, with the results presented in Figure 4. In comparison to the control group, the ethanol group exhibited a significant decrease in fluorescence intensity, indicating damage to the mitochondrial membrane integrity due to ethanol stress. However, upon the addition of arginine, the reduction in fluorescence intensity caused by ethanol stress was partially mitigated, suggesting that arginine intervention aids in maintaining mitochondrial membrane integrity under such stress conditions.

### 3.4. Arginine Facilitated Nitric Oxide Synthesis in Ethanol-Stressed W. anomalus

**Figure 5:**
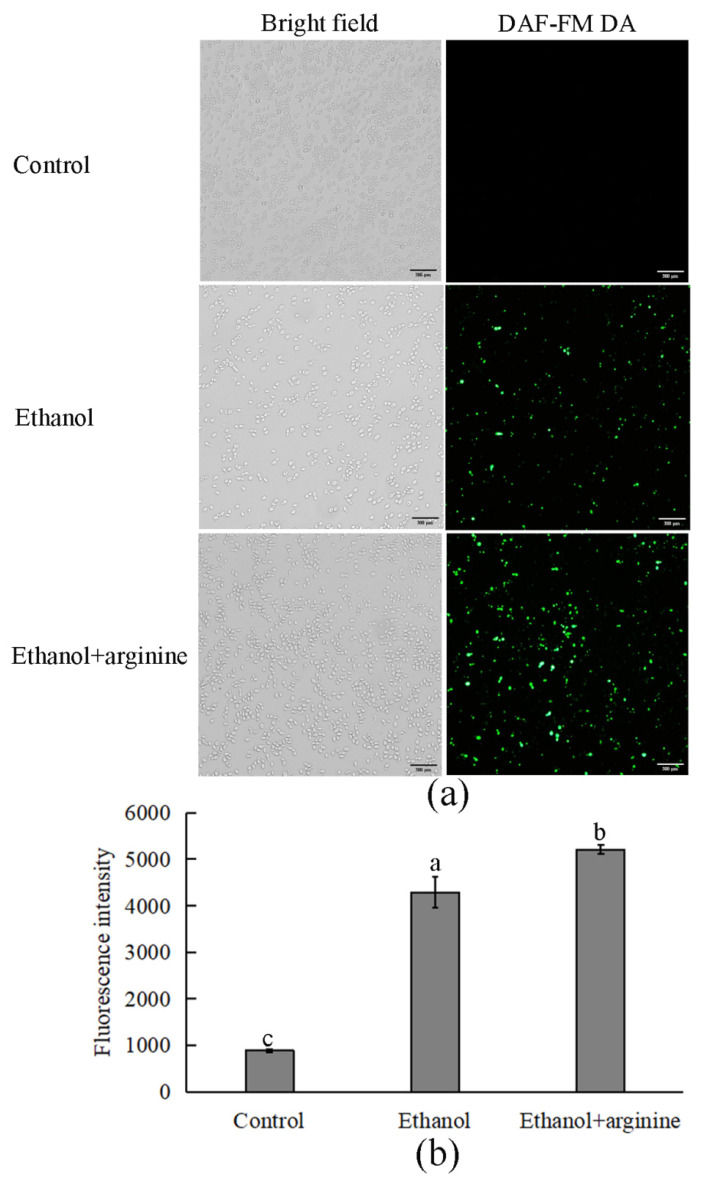
Arginine facilitated nitric oxide synthesis in ethanol-stressed *W. anomalus.* (**a**) Fluorescence images of each group; (**b**) fluorescence intensity of each group. Values of the distinct lowercase letters indicate significant differences (*p* < 0.05). Scale bar = 300 μm.

As a precursor for nitric oxide production, arginine plays a pivotal role in cellular responses to various stressors [20]. To investigate this, nitric oxide levels were quantified using a fluorescent staining technique in the presence and absence of arginine under ethanol stress conditions. The levels of nitric oxide were observed to be extremely low under non-stressed culture conditions, suggesting that minimal nitric oxide was synthesized. However, upon exposure to ethanol stress, we observed a marked induction in nitric oxide synthesis, as evidenced by an increase in fluorescence intensity (Figure 5a,b). This finding suggests that nitric oxide synthesis is activated as a cellular response to ethanol stress. Interestingly, when arginine was added to the culture medium under ethanol stress conditions, we observed a further increase in nitric oxide production. This augmentation in nitric oxide synthesis indicates that arginine not only participates in the nitric oxide synthesis pathway but also potentiates this process in response to ethanol stress, potentially aiding in the cellular adaptation and defense mechanisms of *W. anomalus*.

### 3.5. Arginine Regulated Gene Expression Profiles in Ethanol-Stressed W. anomalus

#### 3.5.1. Identification of DEGs

Comprehensive sequencing analysis was performed to investigate the impact of arginine on the gene expression profiles of *W. anomalus* under ethanol stress. A total of 43,065,443 raw reads, 5,028,819,943 raw bases, 42,245,314 clean reads, and 6,315,356,769 clean bases were obtained for the control group. In the ethanol-stressed group, the numbers were 46,447,271 raw reads, 7,013,537,971 raw bases, 44,885,465 clean reads, and 6,677,724,185 clean bases. Similarly, the ethanol + arginine group yielded 45,249,228 raw reads, 6,832,633,428 raw bases, 44,321,098 clean reads, and 6,630,839,405 clean bases (Table 1). The error rates were 0.02% for both the control and ethanol groups, and 0.03% for the ethanol + arginine group. The Q20 (%) and Q30 (%) values were greater than 90% for all groups. Furthermore, principal component analysis (PCA) revealed distinct clustering of samples from each group within separate confidence circles (Figure S2). Collectively, these data confirm the accuracy and reliability of the sequencing results obtained in this study, thereby establishing a solid basis for identifying DEGs and elucidating the regulatory function of arginine in modulating the gene expression profiles of *W. anomalus* under ethanol stress conditions.

**Figure 6:**
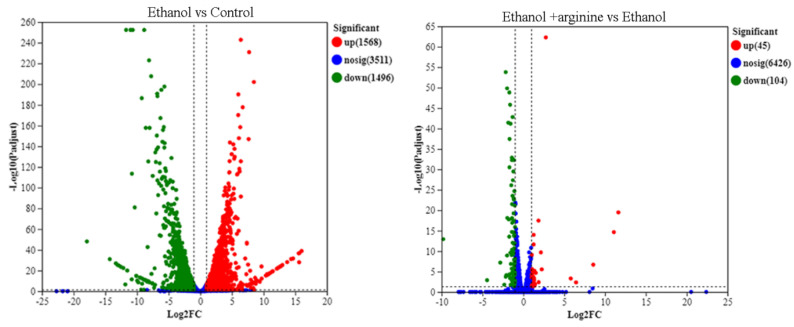
Volcano plot depicting DEGs under ethanol stress and ethanol stress combined with exogenous arginine supplementation.

To gain insights into the effects of ethanol stress and exogenous arginine supplementation on gene expression in *W. anomalus*, the number of DEGs across different groups was compared and visualized as volcano plots (Figure 6). After 6 h of ethanol treatment, 1568 DEGs were upregulated and 1496 were downregulated compared to the control group. However, when ethanol treatment was combined with exogenous arginine supplementation, the number of DEGs was significantly reduced, with only 45 genes upregulated and 104 genes downregulated. These findings suggest that arginine may play a role in modulating the gene expression changes induced by ethanol stress in *W. anomalus*.

#### 3.5.2. KEGG Annotation Analysis of DEGs

**Figure 7:**
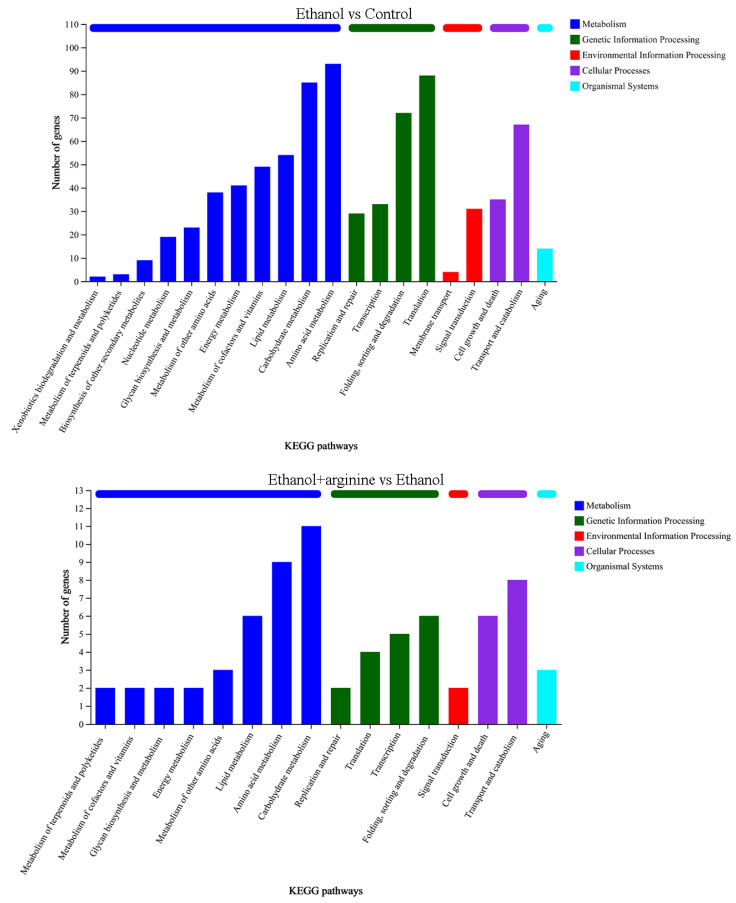
KEGG classification analysis of DEGs under ethanol stress and ethanol stress with exogenous arginine supplementation.

KEGG (Kyoto Encyclopedia of Genes and Genomes) annotation analysis was conducted to gain insights into the functional roles of the DEGs identified in this study. The DEGs were classified into five categories: metabolism, genetic information processing, environmental information processing, cellular processes, and organismal systems. The KEGG annotation results for the DEGs regulated by ethanol stress are presented in Figure 7. Within the metabolism category, the majority of DEGs were annotated to “amino acid metabolism,” followed by “carbohydrate metabolism” and “lipid metabolism.” For the genetic information processing category, most DEGs were annotated to “translation.” Within the environmental information processing and cellular processes categories, the most prevalent annotations were “signal transduction” and “transport and catabolism,” respectively. Only one term, “aging,” was annotated to the organismal systems category.

After exogenous addition of arginine, the metabolism category was dominated by DEGs annotated to “carbohydrate metabolism.” For the genetic information processing category, the most prevalent annotation was “folding, sorting, and degradation.” The annotations for the environmental information processing, cellular processes, and organismal systems categories were similar to those observed in the ethanol stress group, with “translation,” “signal transduction,” and “aging” being the most common, respectively.

Collectively, our findings suggest that ethanol stress primarily affects amino acid metabolism, carbohydrate metabolism, translation, and signal transduction. The exogenous addition of arginine may improve transmembrane transport of metabolites, as the term “membrane transport” was not annotated when cells were simultaneously stressed with ethanol and supplied with exogenous arginine. Additionally, arginine may alleviate the disruptions in amino acid metabolism and carbohydrate metabolism by reducing the number of DEGs in these categories.

#### 3.5.3. KEGG Enrichment Analysis of DEGs

**Figure 8:**
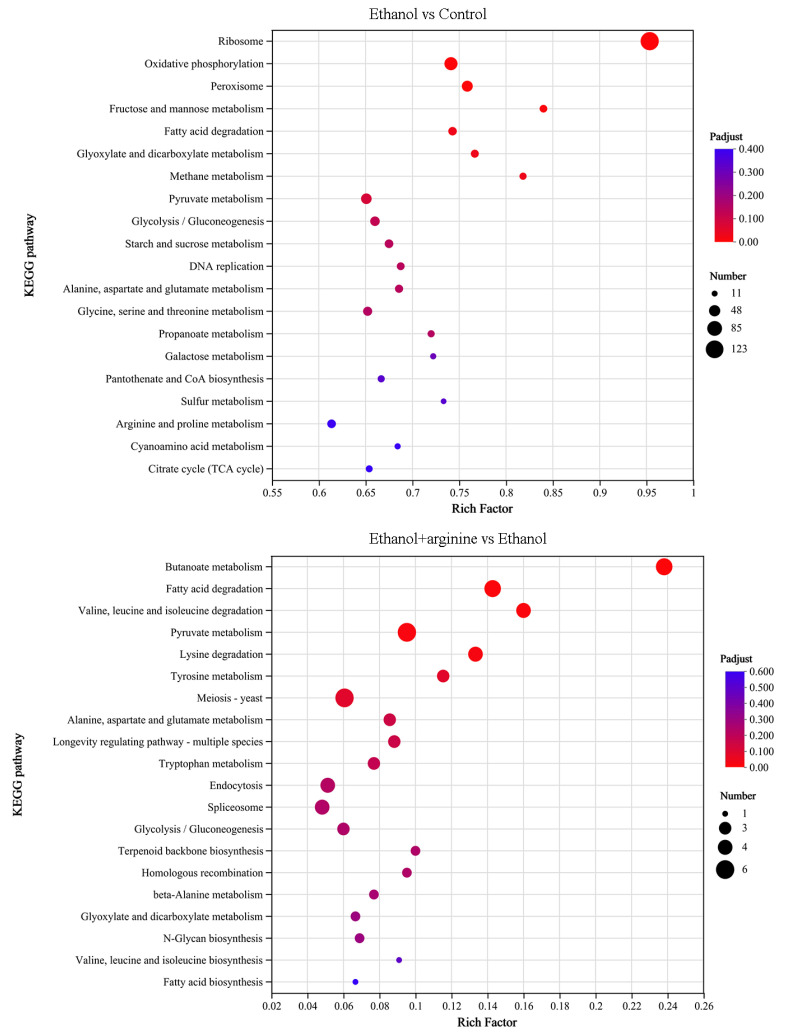
KEGG enrichment analysis of DEGs with ethanol stress or ethanol stress simultaneously supplied with exogenous arginine.

KEGG enrichment analysis were performed to further probe the function of these DEGs, and the results are demonstrated in Figure 8. Upon examination, it becomes evident that the majority of DEGs regulated by ethanol stress were enriched in pathways related to “ribosome” and “oxidative phosphorylation.” However, when arginine was introduced under ethanol stress conditions, the enrichment pattern shifted significantly. The DEGs exhibited enrichment in pathways such as “pyruvate metabolism,” “meiosis-yeast,” “fatty acid degradation,” and “butanoate metabolism.” These findings suggest that arginine intervention alters the metabolic pathways affected by ethanol stress, potentially mitigating some of its adverse effects.

#### 3.5.4. Validation of Transcriptome Sequencing Data by Quantitative Real-Time PCR

To validate the reliability of the transcriptomics data from the present study, the expression levels of eight candidate DEGs—four upregulated and four downregulated—were selected and confirmed using quantitative real-time PCR. The results, presented in Figure S3, indicate that the expression levels of *wa22056* and *wa12004* were downregulated, while those of *wa21665* and *wa106134* were upregulated in the ethanol group vs. the control group. In addition, the expression levels were downregulated for *wa94806* and *wa61681*; in contrast, the expression level were upregulated for *wa19825* and *wa76806* between the ethanol + arginine group and the ethanol group. The expression trends observed by transcriptome sequencing were consistent with those detected by real-time PCR, confirming the reliability of our transcriptome sequencing data.

### 3.6. Arginine Regulated the Metabolite Expression Profiles of Ethanol-Stressed W. anomalus

#### 3.6.1. Identification of DEMs

To assess the quality of the transcriptomic sequencing samples, PCA was initially performed. The results indicate that the samples within each group, both in positive and in negative ion modes, were well clustered within separate confidence circles, suggesting that the repeatability of these samples was stable (Figure S5).

**Figure 9:**
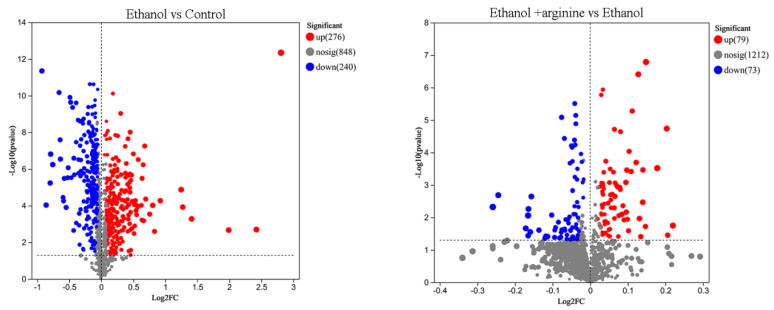
Volcano plot of DEMs with ethanol stress or ethanol stress simultaneously supplied with exogenous arginine.

Subsequently, DEMs were screened, and the results are presented in Figure 9. A total of 516 DEMs were detected between the ethanol group and the control group, including 276 upregulated DEMs and 240 downregulated DEMs, in both positive and negative ion modes. Additionally, 152 DEMs were identified between the ethanol + arginine group and the ethanol group in both ion modes, with 79 DEMs upregulated and 73 DEMs downregulated. Furthermore, the majority of these DEMs were further classified as amino acids, phospholipids, nucleotides, monosaccharides, and carboxylic acids using the KEGG compound classification database (Figure S6).

#### 3.6.2. KEGG Annotation Analysis of DEMs

**Figure 10:**
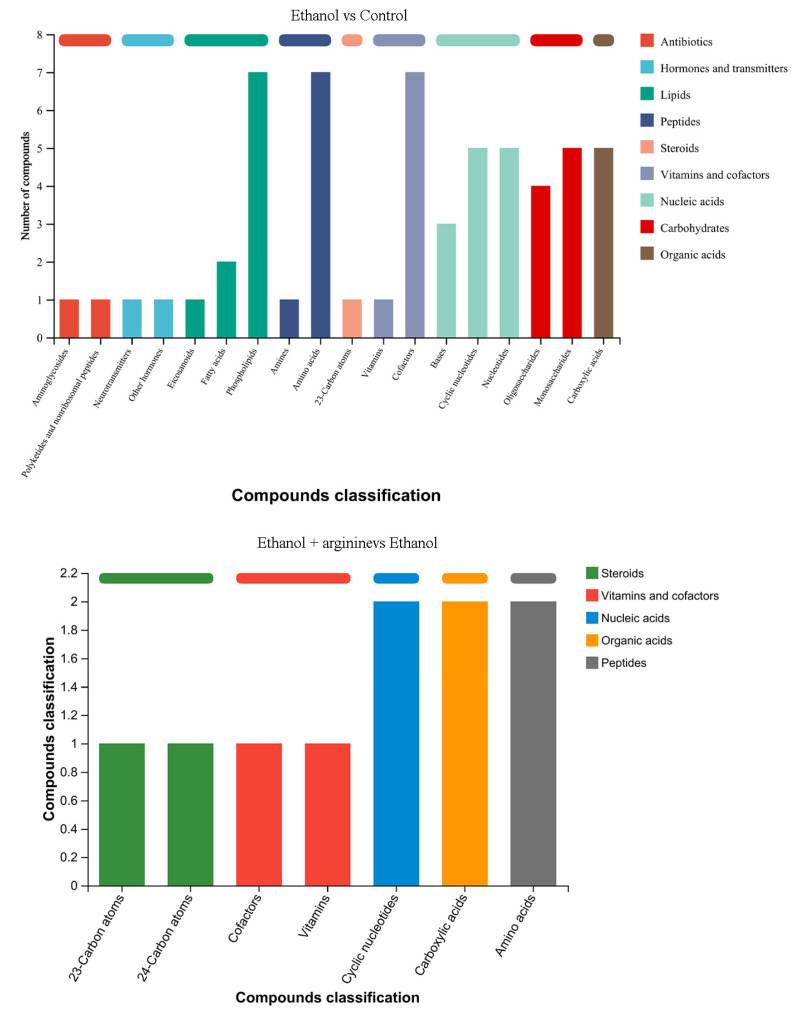
KEGG annotation analysis of DEMs under ethanol stress and ethanol stress with exogenous arginine.

When comparing the DEMs between the ethanol group and the control group, most of the identified metabolites belonged to phospholipids, amino acids, and cofactors (Figure 10). Additionally, cyclic nucleotides, nucleotides, monosaccharides, and carboxylic acids were also identified in higher numbers compared to other compounds.

Following arginine intervention under ethanol stress, the DEMs were further classified into five categories: steroids, vitamins and cofactors, nucleic acids, organic acids, and peptides. Notably, cyclic nucleotides, carboxylic acids, and amino acids emerged as the most prevalent DEMs in this group.

As depicted in Figure S6, the DEMs comparison between the ethanol group and the control group revealed significant enrichment in pathways related to the biosynthesis of cofactors, purine metabolism, nucleotide metabolism, and ABC transporters. Additionally, metabolic pathways such as arginine and proline metabolism, lysine degradation, tryptophan metabolism, and amino sugar and nucleotide sugar metabolism were also enriched. These findings suggest that ethanol stress impacts a wide range of metabolic processes, particularly those involved in nucleotide synthesis and transport, as well as amino acid metabolism.

When comparing the ethanol + arginine group to the ethanol group, the enrichment analysis highlighted pathways such as biosynthesis of cofactors, tryptophan metabolism, glycerophospholipid metabolism, and the biosynthesis of various other secondary metabolites. Notably, biosynthesis of cofactors and tryptophan metabolism were enriched in both treated groups, indicating their consistent involvement in the metabolic response to ethanol stress, regardless of arginine supplementation.

However, glycerophospholipid metabolism and the biosynthesis of various other secondary metabolites were uniquely enriched in the ethanol + arginine group. This suggests that exogenous arginine may specifically influence these metabolic pathways, leading to alterations in the DEM profile that were not observed under ethanol stress alone.

## 4. Discussion

In recent years, non-Saccharomyces yeast *W. anomalus* has attracted considerable research interest for its role in modulating wine flavor profiles. This yeast is frequently studied in mixed fermentations with *S. cerevisiae* across various winemaking processes, including Baijiu, rice wine, and fruit wines [21,22]. When combined with *S. cerevisiae* as mixed fermentation starters or utilized for bioaugmentation in solid-state fermentation during *Baijiu* production, *W. anomalus* has been observed to elevate the content of ethyl acetate, a pivotal compound that profoundly influences the style and quality of *Baijiu* [23,24]. Furthermore, during rice wine fermentation, this aroma-producing yeast strain enhances the wine’s aroma profile by boosting the production of flavor compounds such as esters, free fatty acids, and alcohols, as well as amino acids, ultimately leading to improved sensory scores [25]. Additionally, *W. anomalus* has demonstrated its ability to modulate the flavor parameters of various fruit wines [26]. For instance, mixed fermentation of longan wine through co-inoculation or sequential inoculation with *W. anomalus* and *S. cerevisiae* has resulted in decreased alcohol content and balanced levels of volatile acids, esters, aldehydes, and ketones, contributing to an overall refined wine profile [27].

During the wine brewing process, elevated ethanol levels can exert a multitude of detrimental effects on yeast cells, including the disruption of cellular structures, the induction of heightened ROS production, and the disruption of gene expression and metabolic reactions [3,4]. Consequently, the development of physical, chemical, or biological strategies to mitigate the stress induced by ethanol on yeast and enhance fermentation efficiency is of paramount importance. In this investigation, we have demonstrated that arginine exhibits a range of cytoprotective effects, which are beneficial to yeast cells under ethanol stress. Specifically, arginine reduces ROS production (Figure 2), preserves the integrity of both the cell membrane and the mitochondrial membrane (Figure 3 and Figure 4), fosters nitric oxide synthesis (Figure 5), and regulates gene expression (Figure 6, Figure 7 and Figure 8) and metabolic profiles (Figure 9 and Figure 10). These mechanisms collectively contribute to the improved survival of *W. anomalus* under ethanol stress, thereby enhancing the overall robustness and efficiency of the fermentation process.

ROS, comprising superoxide anions (O_2_^.−^), hydroxyl radicals (^.^OH), and hydrogen peroxide (H_2_O_2_), among others, are byproducts predominantly generated by the electron transport chain in mitochondria during aerobic metabolism [28]. Numerous studies have highlighted the pivotal regulatory role of ROS in diverse cellular processes [29,30]. At low concentrations, ROS stimulate cell proliferation and mitosis; however, excessive ROS levels can impair the cell cycle and induce apoptosis by oxidizing intracellular biomacromolecules such as proteins, nucleic acids, and lipids. Notably, excessive ROS production frequently occurs during fermentation in response to various adverse environmental stressors, including ethanol stress [31]. In this study, we observed a surge in ROS levels in *W. anomalus* exposed to 9% (*v*/*v*) ethanol treatment (Figure 2a). Conversely, upon exogenous addition of arginine, reduced ROS levels were detected, suggesting that arginine possesses the ability to partially eliminate excess ROS from cells (Figure 2b). This ROS scavenging activity likely contributes to the preservation of structural integrity in both the cell membranes and the mitochondrial membranes of *W. anomalus* under ethanol stress (Figure 3 and Figure 4). Nevertheless, the precise mechanism underlying arginine’s ROS scavenging capabilities warrants further investigation to fully elucidate its cytoprotective effects in this context.

Nitric oxide, a small, diatomic gaseous signaling molecule, possesses the unique ability to readily diffuse across cell membranes and reach various cellular compartments [32,33]. Due to its high reactivity, nitric oxide plays a crucial role in modulating cellular responses to a wide array of stressors [34]. Accumulating evidence emphasizes its pivotal function in mitigating the detrimental effects of both abiotic and biotic stresses on plants, encompassing heavy metal stress, temperature extremes, salinity stress, and attacks from pests and pathogens [35,36,37,38]. In yeast species, nitric oxide has demonstrated protective effects in *S. cerevisiae* under copper and heat stress conditions [39,40], as well as in *Schizosaccharomyces pombe* subjected to oxidative stress [41]. In this study, we observed a significant induction of nitric oxide synthesis in *W. anomalus* in response to ethanol stress (Figure 5a). This finding suggests that nitric oxide may play a beneficial role in regulating the adaptive response of *W. anomalus* to ethanol stress. Notably, the exogenous addition of arginine, a known synthetic precursor of nitric oxide, further enhanced nitric oxide synthesis (Figure 5b). This augmentation in nitric oxide production appeared to alleviate the toxic effects of ethanol, indicating a potential cytoprotective mechanism mediated by arginine-induced nitric oxide synthesis in *W. anomalus* under ethanol stress.

Recently, multi-omics techniques, such as transcriptomics, metabolomics, and proteomics, have exhibited robust analytical capabilities and immense application potential in offering comprehensive insights into cellular responses to diverse stresses [42]. Researchers have integrated two or more of these omics techniques to delve into the effects of ethanol treatment at varying concentrations on yeast cell structure and physiological metabolism [43]. For instance, Li et al. [44] utilized RNA-seq and iTRAQ LC-MS/MS techniques to investigate the transcriptomic and proteomic responses of *S. cerevisiae* Sc131 to ethanol stress. They identified a total of 937 DEGs and 457 differentially expressed proteins (DEPs) associated with filamentous growth, sexual reproduction, signal transduction, metal ion regulation, and protein–protein interactions. In our prior research, we investigated the mechanism underlying the ethanol stress response in *W. anomalus* by integrating transcriptomics and metabolomics approaches [8]. Our findings revealed significant impacts on energy metabolism, amino acid metabolism, fatty acid metabolism, and nucleic acid metabolism due to ethanol stress. In the current study, we employed transcriptomics and metabolomics techniques to examine the mechanism behind the alleviating effect of arginine on ethanol stress in *W. anomalus*. Our results indicated that pyruvate metabolism, yeast meiosis, fatty acid degradation, glycerophospholipid metabolism, and the biosynthesis of various secondary metabolites were significantly influenced by the exogenous addition of arginine (Figure 8 and Figure S6). These findings offer novel insights into the molecular mechanisms governing the response of *W. anomalus* to ethanol stress under arginine treatment.

Arginine has attracted considerable attention for its ability to alleviate cellular stress under various adverse conditions [45]. Studies have shown that arginine supplementation can enhance cellular tolerance to stress by facilitating the synthesis of protective molecules such as nitric oxide, which functions as a signaling molecule to regulate stress-responsive genes and antioxidant defense systems [10,11]. For example, in *S. cerevisiae* cells subjected to ethanol stress, arginine has been demonstrated to decrease ROS accumulation, stabilize membrane integrity, and restore mitochondrial function [7]. In the present study, we also observed lower ROS levels and improved mitochondrial membrane integrity in ethanol-stressed *W. anomalus* cells using fluorescence staining methods (Figure 2 and Figure 4). Furthermore, transcriptomics analysis revealed significant enrichment in pyruvate metabolism, fatty acid degradation, and butanoate metabolism, while metabolomics analysis showed a notable enrichment in carboxylic acids and amino acids (Figure 8 and Figure S6). These findings suggest that arginine supplementation improved mitochondrial function by restoring membrane structure integrity.

Additionally, arginine metabolism contributes to the production of polyamines and proline, both of which play crucial roles in maintaining cellular homeostasis under stress conditions [46]. However, the effects of supplying of arginine on the metabolism of polyamines and proline was not fully investigated in this study, and it is essential to deal with this issue in the near future.

## 5. Conclusions

To the best of our knowledge, this study presents the first evidence of arginine’s cytoprotective role against ethanol stress in *W. anomalus*. The protective mechanism involves scavenging excess ROS, preserving cellular and mitochondrial membrane integrity, stimulating nitric oxide production, modulating gene expression and metabolic pathways, and ultimately enhancing cell viability. These findings underscore the potential of arginine as a protective agent for improving ethanol stress resilience in industrial microorganisms. Further research is necessary to evaluate its effectiveness in augmenting ethanol stress tolerance across diverse biological systems.

## Figures and Tables

**Table 1:** Quality analysis of transcriptome sequencing data for each group.

	Raw Reads	Raw Bases	Clean Reads	Clean Bases	Error Rate (%)	Q20 (%)	Q30 (%)	GC Content (%)
Control	43,065,443	6,502,881,943	42,245,314	6,315,356,769	0.02	98.25	94.71	37.133
Ethanol	46,447,271	7,013,537,971	44,885,465	6,677,724,185	0.02	98.24	94.77	40.023
Ethanol + arginine	45,249,228	6,832,633,428	44,321,098	6,630,839,405	0.03	98.21	94.64	38.42

## Data Availability

The original contributions presented in this study are included in the article. Further inquiries can be directed to the corresponding authors.

## Supplementary Materials

The following supporting information can be downloaded at: https://www.mdpi.com/article/10.3390/jof11070499/s1, Figure S1: Results of superoxide anion (O_2_^·−^) detected by NBT staining; Figure S2: Principal component analysis (PCA) results of transcriptome sequencing samples of different groups; Figure S3: Real-time PCR validations of RNA-seq data; Figure S4: KEGG enrichment analysis of DEMs with ethanol stress or ethanol stress simultaneously supplied with exogenous arginine; Figure S5: Principal component analysis (PCA) results of metabolome sequencing samples of different groups; Figure S6: KEGG compound classification of differentially expressed metabolites; Table S1: Primers used in this study.
