# Supplementary material for: The Alleviating Effect of Arginine on Ethanol Stress in Wickerhamomyces anomalus
Source: J Fungi (Basel). 2025 Jul 2;11(7):499. doi: 10.3390/jof11070499 (PMC12299505; doi:10.3390/jof11070499)
Supplement: Supplementary file 1 [file jof-11-00499-s001.zip › jof-3675458-supplementary.pdf]

Supplementary materials

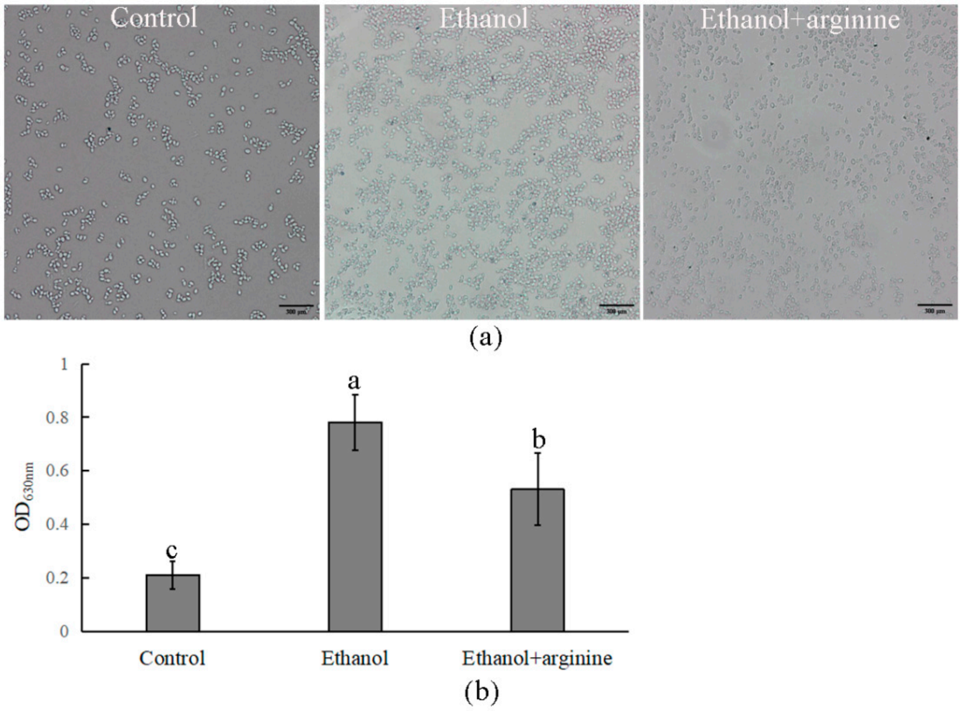

Fig. S1 Results of superoxide anion ($O_2^{\cdot -}$) detected by NBT staining.

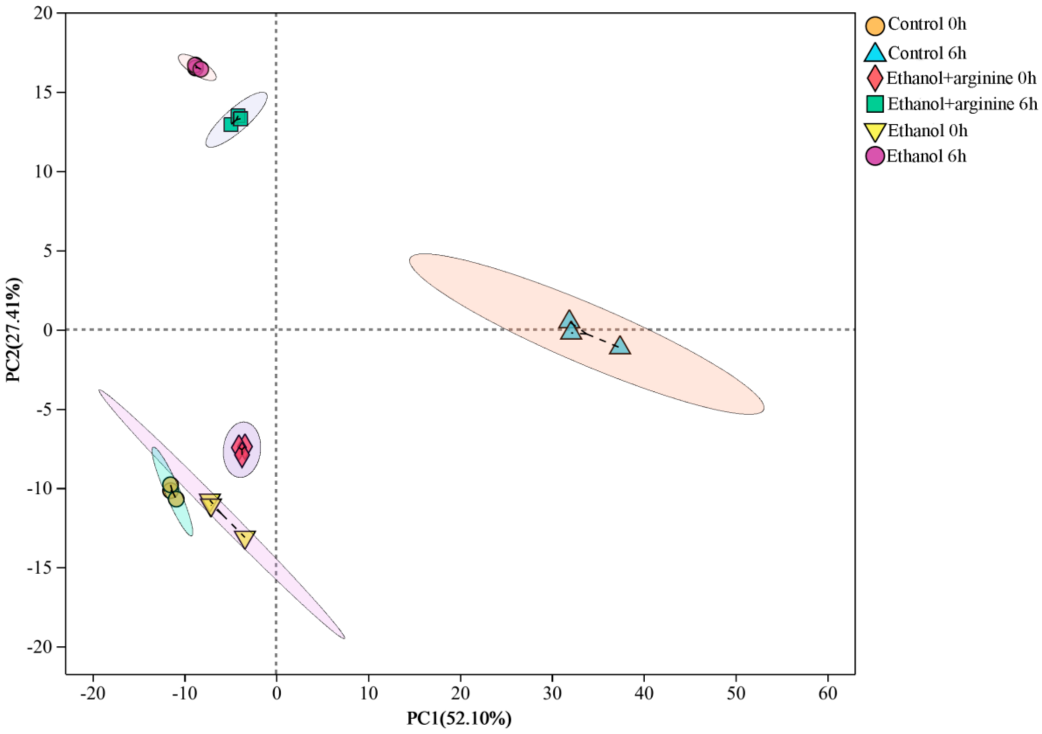

Fig. S2 Principal component analysis (PCA) results of transcriptome sequencing samples of different groups

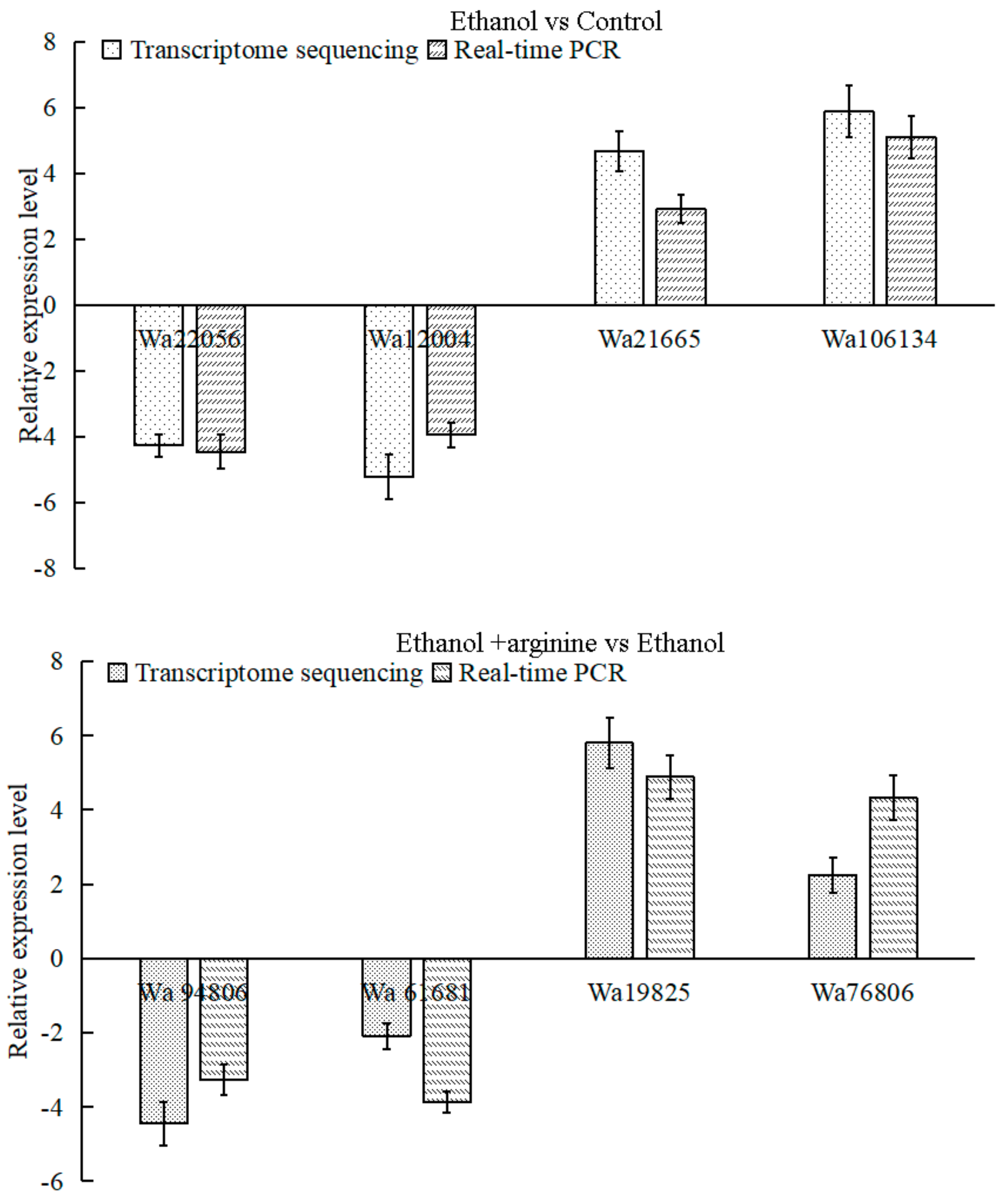

Fig. S3 Real-time PCR validations of RNA-seq data.

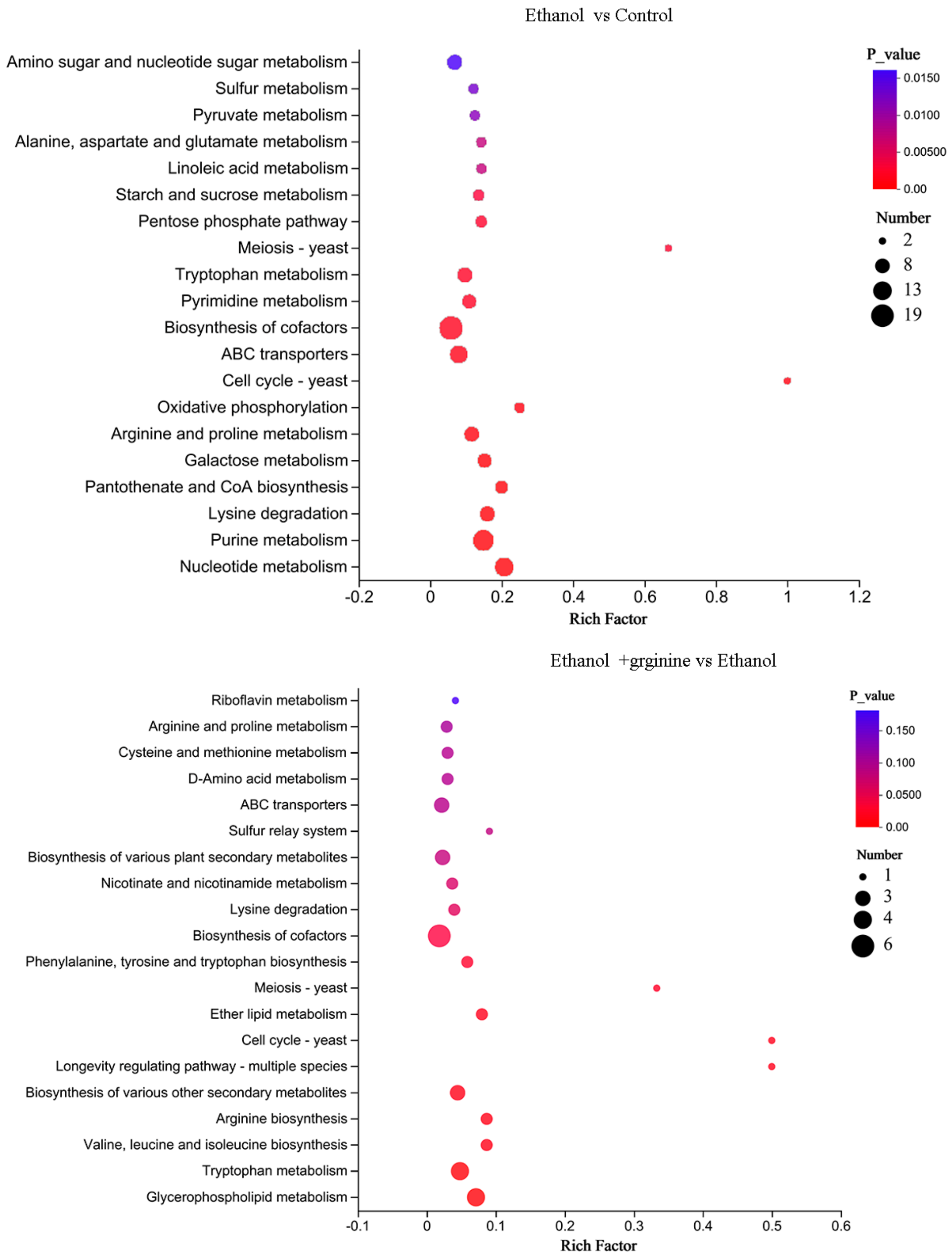

Fig. S4 KEGG enrichment analysis of DEMs with ethanol stress or ethanol stress simultaneously supplied with exogenous arginine.

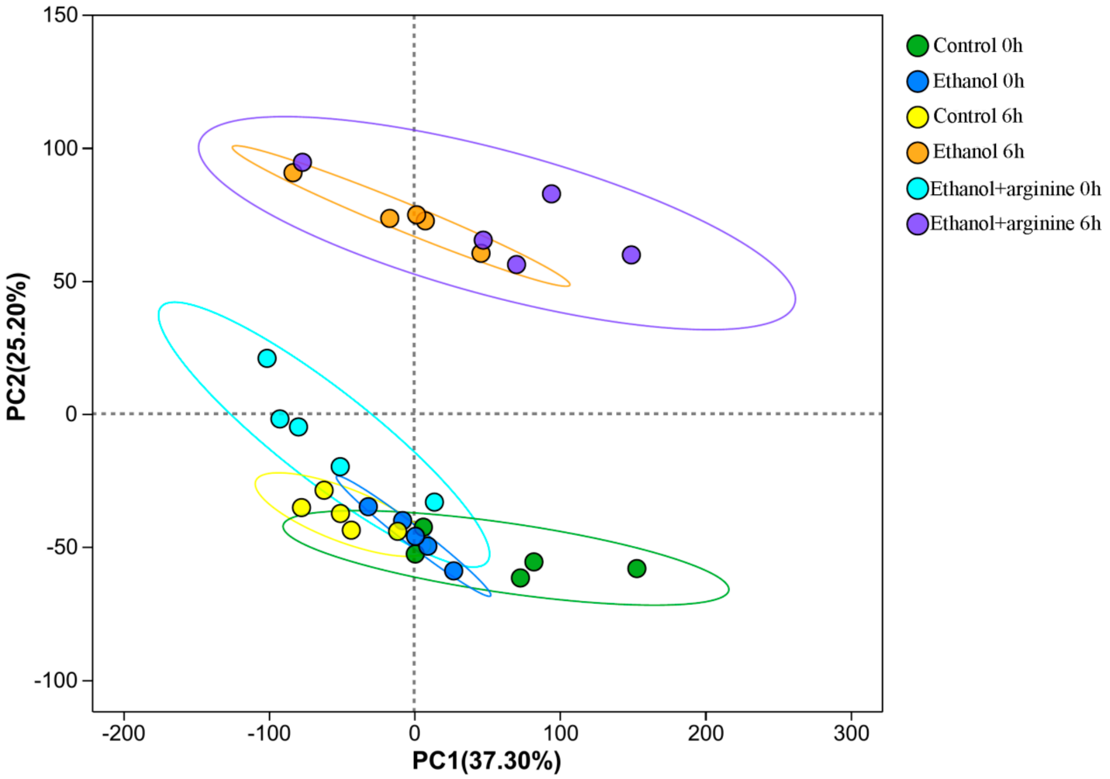

Fig. S5 Principal component analysis (PCA) results of metabolome sequencing samples of different groups

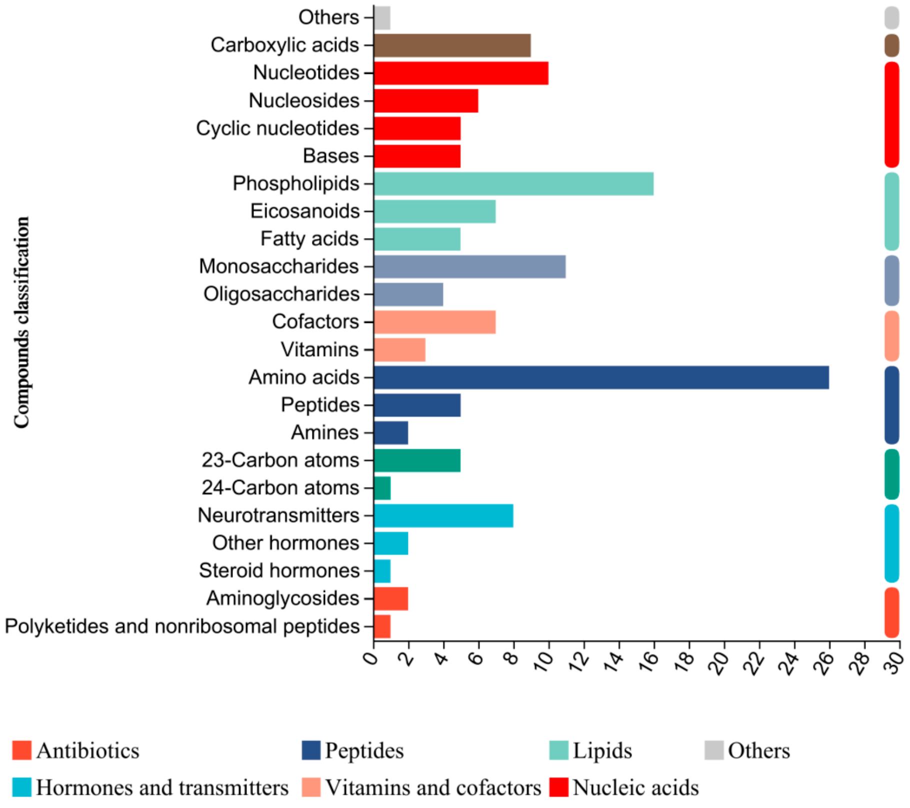

Fig.S6 KEGG compound classification of differentially expressed metabolites.

Table S1.Primers used in this study.

Gene name	Prime name	Prime sequences	PCR product size
Wa22056	22056-F	CGGTAAATGTGGTTATCCAATTAC	204
	22056-R	CTGGAGTGACTTCATTCTTCACT	
Wa12004	12004-F	CAAGACCTGCTTATAACTTATCC	178
	12004-R	CAATGTATAGCCTGGATTTGATC	
Wa21665	21665-F	GTGGTTCAGGTGCTGGTTCAG	156
	21665-R	CCACACCTGAACAATCAGTGTC	
Wa106134	106134-F	GTATTCACTTGCTCAAAGGTTG	146
	106134-R	CTGTGGTTTCCTTATCTAATG	
Wa94806	94806-F	CAGAAGTTGCTCAATTACAAG	168
	94806-R	CACCTGATTTTATTGTACCGA	
Wa61681	61681-F	CATTTCATTATCACTAGCTAATC	178
	61681-R	AGCACAATCATCAGCTGAGGCTGC	
Wa19825	19825-F	GTGCTTCCAGCACTTTGATG	219
	19825-R	GCATGGTGATAAGACTTAAG	
Wa76806	76806-F	CAAGATAATCTTAAACTAACTG	143
	76806-R	GAGATTTTGGAAGGATCAATATC	

Supplementary method

Real-time quantitative PCR (q-PCR) analysis

Total RNA was extracted from the control, ethanol, and ethanol + arginine groups using Trizol reagent (Invitrogen, USA), following the manufacturer's protocols. Following RNA isolation, cDNA was synthesized using the PrimerScript RT Reagent Kit (Takara, Japan) to generate a robust cDNA template for subsequent qPCR analysis. The qPCR reactions were conducted on the Light Cycler 96 detection system (Roche, Germany). The primers employed in this study for qPCR were listed in Table S1. To analyze the qPCR data, the expression levels of the target genes were normalized against the expression of the housekeeping gene actin, which served as a stable reference for gene expression levels. The normalized expression data were then evaluated using the $2^{-\Delta\Delta CT}$ method.
